# AID in Chronic Lymphocytic Leukemia: Induction and Action During Disease Progression

**DOI:** 10.3389/fonc.2021.634383

**Published:** 2021-05-10

**Authors:** Pablo Oppezzo, Marcelo Navarrete, Nicholas Chiorazzi

**Affiliations:** ^1^Research Laboratory on Chronic Lymphocytic Leukemia, Institut Pasteur de Montevideo, Montevideo, Uruguay; ^2^School of Medicine, University of Magallanes, Punta Arenas, Chile; ^3^The Karches Center for Oncology Research, The Feinstein Institutes for Medical Research, New York, NY, United States

**Keywords:** AID, CLL, microenvironment, off-target mutations, SHM

## Abstract

The enzyme activation-induced cytidine deaminase (AID) initiates somatic hypermutation (SHM) and class switch recombination (CSR) of immunoglobulin (Ig) genes, critical actions for an effective adaptive immune response. However, in addition to the benefits generated by its physiological roles, AID is an etiological factor for the development of human and murine leukemias and lymphomas. This review highlights the pathological role of AID and the consequences of its actions on the development, progression, and therapeutic refractoriness of chronic lymphocytic leukemia (CLL) as a model disease for mature lymphoid malignancies. First, we summarize pertinent aspects of the expression and function of AID in normal B lymphocytes. Then, we assess putative causes for AID expression in leukemic cells emphasizing the role of an activated microenvironment. Thirdly, we discuss the role of AID in lymphomagenesis, in light of recent data obtained by NGS analyses on the genomic landscape of leukemia and lymphomas, concentrating on the frequency of AID signatures in these cancers and correlating previously described tumor-gene drivers with the presence of AID off-target mutations. Finally, we discuss how these changes could affect tumor suppressor and proto-oncogene targets and how they could be associated with disease progression. Collectively, we hope that these sections will help to better understand the complex paradox between the physiological role of AID in adaptive immunity and its potential causative activity in B-cell malignancies.

## Introduction

In general, cancers progress by the emergence of subclones with additional, distinct genomic aberrations not recognized in the initial tumor. These subclones possess advantages in cell survival and/or growth. It is still debated whether these more aggressive cell variants are present from the beginning of the neoplasm, develop afterwards, or are induced or selected by therapy. Regardless, the key concept is that these variants must be generated to lead to disease progression and therapeutic resistance.

DNA abnormalities can result from several processes, either inherent to the cell type being considered and/or induced by environmental insults. In this document, we concentrate on the ability of activation-induced deaminase (AID) to generate such genetic variants, focusing on the specific leukemic subsets in which AID protein is upregulated. Finally, we approach the pathological role of AID and the consequences of its actions on the development, progression, and therapeutic refractoriness of chronic lymphocytic leukemia (CLL) as a model disease for mature lymphoid malignancies.

## Activation-Induced Deaminase in Normal B Lymphocytes and Its Effects on Normal B-Cell Biology

### AID as a Key Molecule in the Adaptive Immune Response

The immune system of vertebrates is unique because the antigen-specific receptor expressed by lymphocytes, which initiates cascades leading to activation of the adaptive immune system, is not the product of a single germline inherited gene. Rather, receptors are generated somatically during cell ontogeny from genes scattered at a particular locus (1). Specifically, an individual B lymphocyte acquires the capacity to respond to external antigens (Ags) but not to self-antigens by a complex and regulated tolerance mechanism. Humoral immunity depends on the production of immunoglobulins (Igs) capable of recognizing the full range of these Ags with high affinity. The generation of this diversity is linked to three different modifications in the genes encoding the Igs: (a) *genetic recombination*, which takes place on the genes encoding the variable (V), diversity (D) and junction (J) regions. This recombination gives rise to the formation of the variable domain of an IG, which will then be associated with the constant region Cμ to establish the first repertoire of IgM-type Ig (2). This event occurs in the fetal liver and in the bone marrow and is independent of the interactions of the B cell with Ag and/or T lymphocytes.

The following two steps take place when B cells meet Ag through the B-cell receptor (BCR). After this exposure, B lymphocytes accumulate in secondary lymphoid organs (SLO) in which two other genome modifications take place: (b) *somatic hypermutation (SHM)* occurs in the variable domains of Ig heavy chains (VH) and light chains (VL) introducing point mutations mainly in the complementary determining regions (CDRs) of VH and VL domains (3), and (c) *class switch recombination (CSR)*, which is also dependent on the Ag and takes place at “pre-switch” regions causing the deletion of portions of the IgH (switch regions) located upstream of each Ig isotype locus, thereby permitting the assembly of the variable domain (VDJ) to the constant domain of a heavy chain (CH) located downstream. Through this process, the Ig effector function exerted by the CH domain is altered, giving rise to the expression of different Ig isotypes (IgG, IgA, and IgE) (4). Isotype-switched Igs more readily leave the circulation and enter solid tissues and eliminate foreign insults. Both SHM and CSR take place in SLO and are Ag- and T cell-dependent (5).

### AID Structure and Function

AID is coded by the *activation-induced cytidine deaminase* (*aicda*) gene that in humans is located on chromosome 12p13 and is a member of the APOBEC (apolipoprotein B mRNA-editing enzyme, catalytic polypeptide) family of deaminases. Because the two genes, APOBEC-1 and AID, are found at the same portion of the same chromosome, it is assumed that they came about from a duplication event (6). AID is a molecule of 198 amino acids with a relative molecular size of 24 kDa and has a 34% identity with APOBEC-1 at the amino acid level (7).

**Figure 1:**
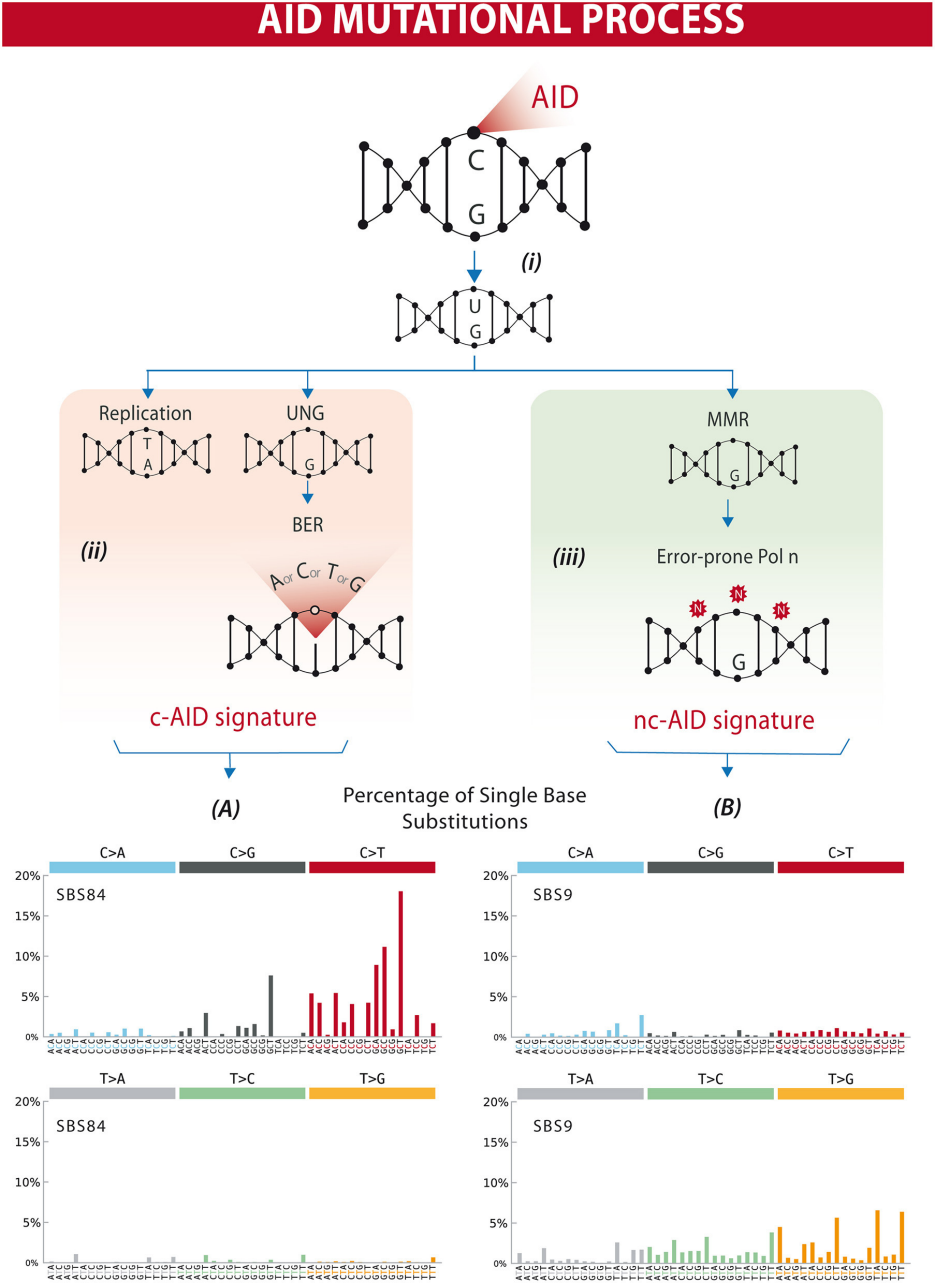
Differential processing of AID lesions. **(i)** AID deaminates cytosine residues on single-stranded DNA that is exposed during transcription, converting C (cytosine) into U (uracil). **(ii)** The U-G (guanine) mismatch can be processed through different pathways. Either by replication that will result in a C>T transition or by uracil DNA glycosylase (UNG) followed by base excision repair (BER) resulting in C>T/G/A substitutions or homologous repair. This will often result in a mutational profile known as canonical AID signature (c-AID). **(iii)** On the other hand, mismatch-repair proteins (MMR) can also recognize and process AID-induced lesions. Exonucleases resect the abasic sites, which are followed by error-prone polymerase repair. This processing often results in a mutational profile similar to the non-canonical AID signature. Lower panels **(A,B)** depict mutational profiles using the conventional 96 mutation type classification. This classification is based on the six substitution subtypes: C>A, C>G, C>T, T>A, T>C, and T>G (all substitutions are referred to by the pyrimidine of the mutated Watson–Crick base pair). Each of the substitutions is examined by incorporating information on the bases immediately 5′ and 3′ to each mutated base generating 96 possible mutation types (6 types of substitution × 4 types of 5′ base × 4 types of 3′ base). Mutational signatures are displayed and reported based on the observed trinucleotide frequency of the human genome, i.e., representing the relative proportions of mutations generated by each signature based on the actual trinucleotide frequencies of the reference human genome version GRCh37. **(A)** SBS84 is found in clustered mutations in the immunoglobulin gene and other regions in lymphoid cancers. **(B)** SBS9 may be due in part to mutations induced during replication by polymerase eta. Mutation frequencies were retrieved from the Comic Catalog v3.1 (cancer.sanger.ac.uk) (13).

The AID gene (*Aicda*) was discovered by a subtraction technique using Ig isotype switch-induced and uninduced derivatives of a murine B-cell line (7). The fact that AID has a catalytic cytosine deamination domain highly conserved among all members of the APOBEC family led to the recognition that the physiological role of AID in B lymphocytes is the induction of mutations and deletions of segments of DNA (8–11). AID can deaminate deoxycytidine in a single-stranded DNA, thus converting it to deoxyuridine (12). This is already a mutagenic lesion causing a C:G to T:A base change after replication. Processing of the uracil by base-excision repair (BER) and mismatch repair (MMR) enzymes leads to the broader spectrum of point mutations characterizing SHM, and to DNA double strand breaks, which are necessary intermediates in CSR [Figure 1 and reviewed in (14, 15)].

Similar to APOBEC-1, AID has cytidine deaminase activity *in vitro* that is inhibited by tetrahydro-uridine, a Zinc ion chelator (7). Despite the fact that, by analogy with APOBEC-1, initially AID was proposed to deaminate cytidines in specific RNAs, no evidence showing this action was documented. However, AID does display a mutational preference for small RNA genes suggesting a putative role for RNA in its recruitment (16, 17). Rather, the DNA deamination model proposing that AID promotes antibody diversity by deaminating deoxycytidine (dC) to deoxyuridine (dU) within Ig genes is currently accepted (18). AID acts on single strand DNA (ssDNA) by inducing multiple deaminations per binding event while remaining bound to the same ssDNA (19, 20). A detailed review of the mechanisms explaining AID activity and the biochemical, biophysical, and structural characteristics of AID activity are reviewed in Feng et al. (15) and Methot and Di Noia (21).

Structure–function relationships of AID have been probed by different *in vitro* experiments focused on natural and engineered mutations in the protein. Interestingly, these data underline differential roles for the N-terminus of AID, which appears to be involved in SHM but not in CSR, whereas the C-terminus appears to selectively initiate CSR (22–24).

Four splice variants of *Aicda* have been identified that remove portions of the C and N termini. Detailed analyses of the expression of these variants have indicated that human centroblasts, the cells that are actively producing AID and undergoing SHM, express only full-length *Aicda* messenger, whereas centrocytes, which are re-expressing membrane Ig and undergoing antigen selection to identify and preserve high-affinity antigen binders, produce full-length and spliced forms of the mRNA (25). Since AID functions as a dimer, these findings suggest a potential inhibitory and amplificatory role for the splice variants, since they could heterodimerize with the full-length form and alter its functional capacities.

Finally, an experiment of nature highlights the importance of AID in CSR and SHM. Hyper-IgM syndrome, type 2, is an autosomal recessive disease caused by mutations in *aicda* (11). People with this syndrome have elevated levels of serum IgM and markedly diminished levels of switched Ig isotypes, consistent with a defect in CSR. Additionally, their Ig molecules do not develop V region mutations, indicating the inability to carry out SHM. Inactivating *aicda* in the murine B cell line used to identify AID leads to similar functional defects (8).

### Molecular Actions and Targeting of AID

AID is a highly efficient DNA mutator, based on the much greater frequency that it alters Ig loci (~10^−4^-10^−3^/base pair/generation) compared to mutations occurring spontaneously in the genome (~10^−9^) (26). Because of its potency, AID's actions need to be tightly regulated. This is accomplished at the transcriptional, post-transcriptional, and post-translational (27–35) levels as well as by its location, which is predominantly cytoplasmic (36, 37).

Additionally, its actions are normally rigorously restricted to Ig loci of B lymphocytes. This is done in a lineage specific and a stage-specific (activated B cell) manner. Restriction to B lymphocytes is controlled primarily by lymphoid-specific transcription factors (38–42). Once directed to the Ig loci of B cells, AID directs its action to the variable (V) and switch (S) regions. At the V region, mutations start shortly downstream of the promoter and proceed for ~2 kB (43). At S regions, mutations are found downstream of the intronic promoter and extend ~4–6 Kb beyond (44). Of note, AID-mediated mutations are rarely found in the C region (45, 46).

AID activity is characterized by cytidine deamination, and repair of U:G lesions may result in mutations within defined trinucleotide signatures. Direct replication over the AID-induced lesions or removal of the uracil by UNG (uracil DNA glycosylase) followed by replication accounts for the mutations of the canonical AID signature (c-AID) (C to T/G mutation at RCY motifs, R = purine, Y = pyrimidine) (47). Processing of the AID-induced lesions by the error-prone DNA polymerase η may result in A>C transversions at WA motifs (W: A or T, A), earlier described as the non-canonical AID signature (nc-AID) (48) (Figure 1). The signature generated by polymerase η repair could reflect either an initial AID-induced lesion or the consequence of other mutagenic events, and therefore the nc-AID signature may be less informative of AID activity than the RCY motif (47, 49, 50). COSMIC analyses on tumor samples in general also demonstrate that this signature is less representative than the classical c-AID signature (Figure 1). A third pattern, recently described in association with AID-mediated CpG-methylation dependent mutagenesis, is characterized by C>T transitions at RCG motifs (49, 51).

AID preferentially targets cytidines (C) at WRC (W = A/T, R = A/G) hotspots (52–54), known targets of SHM (55–60). The resulting mutations are the consequences of the enzyme's direct action as well as from the repair mechanisms (BER or MMR) that follow to correct or not the change. These mutations are primarily transitions rather than transversions (~2:1) (Figure 1). Additionally, the overlapping AID hotspot, WGCW, has a markedly enhanced propensity to be deaminated (61). If overlapping hotspots in CDRs are not altered, there is a significant decrease in the mutation frequency throughout the V region (61, 62).

SHM is focused to DNA sequences that are being transcribed (63–65), occurring most effectively at pause sites (66). Consistent with AID acting on ssDNA is the finding that highly transcribed, non-Ig genes can be mutated (*Bcl-6, Bcl7, Myc, Pax-5, Pim-1, Rhoh, S1pr2, Socs1, CD95*, and *mir142*) (26, 67). This occurs in normal B cells (68–79) as well as in B-cell lymphomas (80–82).

Thus, in some instances, restricting AID's mutational and deletional functions to the Ig loci is comprised, resulting in DNA alterations at other sites in the genome. This aberrant action has been found in cancers not only of the B-lymphocyte lineage (83, 84) but also in other lymphoid (85) and non-lymphoid types (86, 87). Moreover, we (88) have reported data consistent with this occurring in CLL, which will be discussed in detail below.

### Normal B Lymphocytes Expressing AID

AID is expressed in activated B lymphocytes, specifically in cells undergoing germinal center activation (centroblasts) (7, 89). Based on finding the gene upregulated upon immunization and finding it in B lymphocytes localized in germinal centers, AID was shown to be crucial in murine and human germinal center B cells, although it can be found in extrafollicular B cells (38). AID can be upregulated in mature B lymphocytes in T-cell-dependent and T-cell-independent manners. The former is carried out by CD40–CD40L interactions in the presence of IL-4 (90). T-cell-independent stimulation can be achieved with TLR7 + IL-4 (91) and by dual engagement of TLRs and the BCR (92–94). Both stimuli upregulate AID and lead to CSR and/or SHM. Moreover, a combination of T-dependent and T-independent stimuli do the same. Notably, in this setting, the combination can reduce the level of signal needed to accomplish AID expression and action (95).

However, the effects of BCR engagement on the production of AID by mature murine B cells stimulated *in vitro* appear to be time dependent. For example, B cells stimulated T-independently by LPS + IL-4 upregulate AID levels dramatically by 48 h. However, engaging the BCR at that point reduces AID levels to essentially baseline within ~ 4 h (96). This fall is due to a block in transcription. The rapidity and efficiency of BCR engagement in downregulating AID is probably aided by the very short half-life of the enzyme. This fall in production is consistent with a negative feedback loop initiated in an activated cell that has achieved enhanced affinity for the stimulating antigen through V region mutations induced by AID.

Finally, although in general AID is found in activated mature B lymphocytes, several reports suggest that it can be found in less mature, developing (immature/transitional 1) B cells (93, 97–100). These young cells can undergo CSR to produce non-IgM antibodies (93, 101, 102) and SHM to generate higher-affinity antibodies (97). Expression of AID in these cells can be constitutive (93, 97–99) or induced by CD40 (103, 104) and TLR (93, 94) signaling.

## The Role of AID in Cancer

AID-generated uracils are recognized by the uracil-DNA glycosylase (UNG) or the MMR heterodimer MSH2/MSH6 (105). The processing of these uracils produces double-strand breaks, which are the substrates of the end-joining mechanisms that complete CSR by joining two separate S regions (106). Although AID was originally believed to specifically target Ig gene V and S regions, it has become evident that AID also deaminates other genes, which, unless faithfully repaired, can be oncogenic (107). UNG and MSH2/MSH6 modulate the mutagenic capacity of AID either by initiating error-free BER and MMR, respectively, or by triggering mutagenic repair (26).

The first evidence about the oncogenic role of AID was supported by mice models in which constitutive *Aicda* gene expression was forced (108). AID-transgenic mice show tumors developed in various cells, in which mutations accumulate in non-Ig genes, including proto-oncogenes (108, 109). However, the number of gene AID that actually targets is not clear (18). Using AID chromatin immunoprecipitation (ChIP), Yamane et al. (67) showed that AID binds to thousands of genes in activated B cells but mutates only a fraction, although these data have been contested (110). So, although the number of non-Ig genes targeted by AID remains elusive, it is clear that AID can initiate chromosomal translocations or point mutations, some of which can be oncogenic (74, 111).

In a normal setting, DSBs are promptly repaired; for instance, homologous recombination prevents widespread DNA breaks by AID (112). Still, since the main factor influencing the rate of translocations is the formation of DSBs, continual localized DNA damage by AID probably favors recurrent translocations (74). A prime example is the *IgH/c-Myc* translocation typical of mouse plasmacytoma models and a hallmark of Burkitt's lymphoma in humans (72, 113). C-myc transgenic animals develop pre-B lymphomas or B lymphomas without SHM, whereas ubiquitous AID transgenic overexpression is sufficient to cause T cell lymphomas, lung adenomas, and adenocarcinomas (108).

Endogenous levels of AID are sufficient to predispose B cells for transformation. This has been demonstrated in IL-6 transgenic or pristane-induced plasmacytomas, in which AID is crucial for the creation of the *IgH/c-Myc* translocation (114, 115). Additionally, similar experiments indicate the importance of AID for diffuse large-cell lymphoma (DLBCL)-like malignancies in the Iμ-BCL6 transgenic mouse model (31). Interestingly, AID deficiency reduces the incidence of B lymphomas, but not pre-B lymphomas, whereas this deficiency also prevents GC- and post-GC-derived lymphomas, but not marginal zone lymphoma development, in Iμ Bcl6 transgenic mice (109). These results indicate that AID is mainly involved in tumorigenesis in mature, activated B cells (18).

AID is expressed physiologically in GC B cells (8) and accordingly in GC-derived human B-cell lymphomas, such as diffuse large B-cell lymphoma (DLBCL), follicular B-cell lymphoma (FL), and Burkitt lymphoma (BL), which express AID constitutively (116–119). While aberrant SHM in normal and lymphoma B cells affects many proto-oncogenes and tumor suppressors (*Myc, Ig alpha, Pax-5, Bcl-6, Rhoh*, and *Pim-1*) (80, 120–122), presently no direct evidence has been published relating an AID DNA mutation signature with these off-target mutations.

AID is consistently expressed not only in neoplastic cells in Hodgkin's lymphoma but also in many other human hematological malignancies including CLL (123–125), B-cell acute lymphoblastic leukemia (126), mantle-cell lymphoma (127), and in some cases of MALT lymphoma, which derives from marginal zone B cells in mucosa-associated lymphoid tissue, of immunocytoma, which is derived from plasma cells (117, 119), and of hairy cell leukemia, which derives from memory cells (128). These studies indicate that AID can be expressed not only in GC-derived B-cell lymphomas but also in leukemias and lymphomas originating from B cells at various stages of differentiation (18). In addition, AID expression can be found in a number of non-B cell malignancies including epithelial cancers such as *H. pylori*-associated gastric cancer (129), hepatocellular carcinomas (130, 131), and lung carcinomas (132). AID expression can be especially problematic in chronic diseases, where even a small but continuous level of AID activity could lead to selectable genetic mutations over time, giving rise to more aggressive tumors and treatment resistance.

## Chronic Lymphocytic Leukemia: The Role of Microenvironmental Signaling on Intraclonal CLL Fractions

CLL is characterized by progressive accumulation of monoclonal B-lymphocytes expressing CD5 and CD23 molecules and characteristic low amounts of surface membrane Ig and CD79b molecules (133). Interestingly, in this leukemia, the mutational profile of VH immunoglobulin genes (IgHV) divides patients into two categories (134, 135) that differ dramatically in prognosis (136, 137). Patients expressing mutated IgHV (M-CLL) develop a more indolent disease, whereas IgHV unmutated (U-CLL) patients display a more aggressive disease that is often unresponsive to treatment. Although CLL remains an incurable disease, very important progress in this area of knowledge has been recently achieved. The accumulation of mature B-cells, which have escaped programmed cell death and have undergone cell cycle arrest in the G0/G1 phase, is characteristic of CLL. In contrast with these *in vivo* features, apoptosis occurs after *in vitro* culture, suggesting an important role of the microenvironment on CLL cell survival (138, 139). CLL is the prototype of a cancer where both genetic and microenvironmental factors promote the onset, expansion, and progression of the disease (140, 141). Although classically CLL was considered a disease of accumulation, new data indicate that CLL expansion is a dynamic process in which cell proliferation compensates for the cell loss from death (142, 143) and that accumulation of the malignant cells reflects a balance between the effects of cell proliferation and death (144). Moreover, the balance between cell proliferation and cell loss appears to vary in different subsets of the disease, which have been defined based on the cell genotypic and phenotypic features (143, 145). The seminal hypothesis postulating that phenotypic cell heterogeneity could exist within the tumor clone of the same CLL patient was first explored two decades ago. The notion that the leukemic clone could hold CLL cells either phenotypically activated or kinetically resting leads to key questions in the CLL biology: (a) Are there distinct subpopulations within the tumor clone? (b) Does the microenvironment favor the development of proliferative CLL subpopulations? (c) Which microenvironmental elements influence the malignant clone and which molecular pathways do they utilize? (d) Is the microenvironment affecting the relationships between proliferation and extended survival?

Evidence for the important role of the BCR in CLL pathogenesis is given by the fact that the mutational status observed in BCR sequences divides CLL cases into two subsets (134, 135), and this is one of the strongest predictors of disease outcome (136, 137). In addition, BCR signaling has been postulated to play a role for CLL trafficking and interaction with the stromal microenvironment (146). Increasing evidence suggests that CLL cells in lymph nodes (LN) and bone marrow (BM) interact with stromal cells and thereby receive proliferative and survival signals. Disease prognosis and evolution is probably related to the consequence of this interaction. In line with this hypothesis, CLL cells residing in LN show increased proliferation when compared to leukemic cells in the bone marrow (BM) and peripheral blood (PB) (147) and have a gene expression profile compatible with activated B cells (148).

Pseudofollicular proliferation centers (PCs) are classical anatomical structures in CLL patients similar to those observed in inflamed tissues of patients with chronic autoimmune/inflammatory disorders. These PCs are composed of a complex mixture of T cells, monocyte-derived cells, and stromal cells that provide pro-survival signals and form suitable niches for tumor growth (149, 150). CLL cells in PCs can have higher levels of the proliferative marker Ki-67 (151). Microenvironmental signals in additional to BCR that appear to be delivered in tissues important for the crosstalk between CLL cells and their microenvironments involve CD40 (138), TLR (152), B-cell activating factor receptor (BAFFr), and transmembrane activator CAML interactor (TACI) (153). However, the relative, individual pathogenetic influences of each molecule are unclear, as it is unknown to what extent they cooperate with the BCR stimulation in different patients.

During recent years, a variety of novel kinase inhibitors targeting various components of the BCR signaling pathway have been designed. These mainly inhibit phosphoinositide 3'kinase (PI3K), Bruton's tyrosine kinase (BTK), and spleen tyrosine kinase (SYK). Each of these shares a pattern of response resulting in nodal reduction and increased lymphocytosis, thus reflecting microenvironmental modulation (154). These new drugs affecting the signaling activation pathway have generated significant promise by targeting the proliferative pools existing in BM and LN and inducing release from and preventing re-entry to survival niches, thus bringing us closer to curing the disease.

### Intraclonal CLL Fractions as a Marker of Disease Progression

The presence of proliferating and accumulating clonal CLL cells inside of the tumor clone in the same patient was postulated by Caligaris-Cappio (155), and this was confirmed in 2005 by Messmer et al. (143). In the latter, CLL patients drank deuterated water (^2^H_2_O), and ^2^H incorporation into the DNA of newly divided CLL cells was measured. Collection and analysis of these data indicated that the leukemic cells of each patient had definable and substantial birth rates, varying from 0.1 to 1.0% of the entire clone per day. These findings were confirmed and extended by others (156, 157). More importantly, the former suggested that those patients with higher proliferation rates experienced a more progressive disease than those with lower birth rates (143), and this was confirmed in a larger, independent study (158).

**Figure 2:**
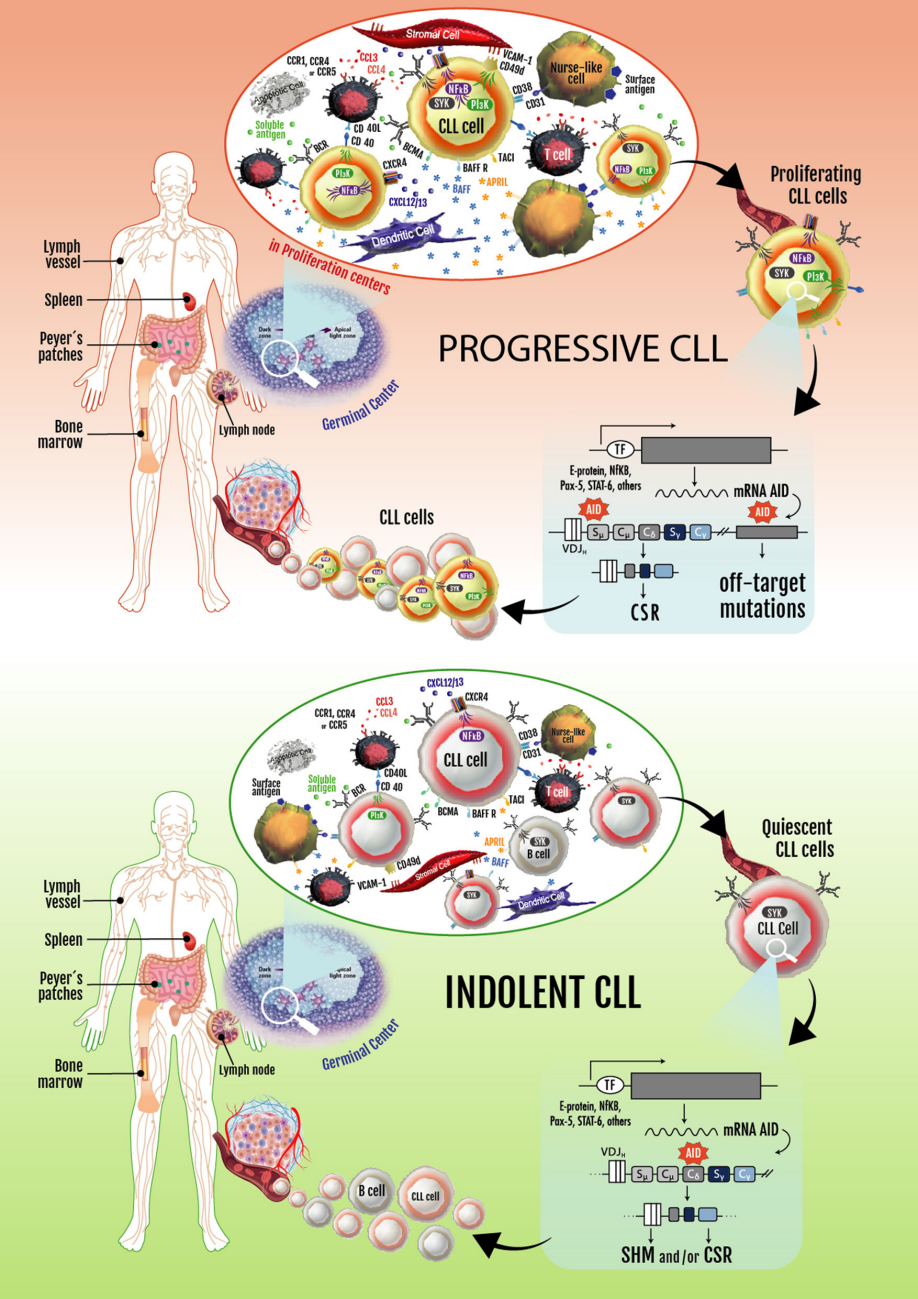
Progressive and indolent CLL landscapes based on microenvironmental influences. In patients with progressive disease, an activated microenvironment continuously nourishes leukemic cells by maintaining proliferative fractions that can express AID. Proliferating cells overexpressing AID are susceptible to novel DNA lesions (many of them in non-Ig genes), establishing clonal and subclonal entities before and/or after treatment. These can lead to CLL progression and/or therapy resistance. Some of the leukemic cells dividing in proliferation centers leave the tissues and move into the blood. These circulating cells must return to lymphoid tissues to receive survival signals. If not, they eventually die. Cycles of these two events overtime lead to increased numbers of circulating AID^+^ leukemic B cells, which is a hallmark of progressive CLL. In patients with indolent disease, microenvironmental signaling is similar to that taking place in normal GCs, with CLL cells becoming physiologically activated and AID expression and non-Ig genes mutations being better controlled.

Based on these observations, two key concepts in the CLL biology were established: (1) CLL is not a static disease but a disease of proliferating and over-surviving pools, and (2) the balance between these subsets explains, at least in part, the heterogeneous clinical outcome of CLL patients (144, 146). These two concepts are exemplified in Figure 2.

In the ensuing years, efforts have been made to characterize the proliferative compartment of the leukemic clone considering that the study of this fraction could lead to new therapeutic targets in CLL. Initial studies focused on leukemic cells expressing CD38 (CD38^+^), a molecule involved in signaling and activation that also serves as a prognostic marker in the disease. This identified a close association between the expression of CD38 expression and of increased Ki-67 and ZAP-70 levels, suggesting that the CD38^+^ fraction contains more activated members and could more frequently enter the cell cycle than the counterpart CD38^−^ fraction (159). However, and despite these activation/proliferation differences, both CD38^+^ and CD38^−^ fractions have similar telomere lengths, suggesting that CD38 expression is a temporal and dynamic marker of an activated B-cell status instead of a specific marker of the proliferative fraction in CLL. As previously mentioned, CD38 expression is also a prognostic marker in CLL in which a correlation with poor prognosis was described for those patients with higher percentages of CD38^+^ cells (>/=30% of the leukemic clone) (136). The fact that CD38 is expressed in a high percentage of leukemic cells in IgHV-unmutated (U-CLL) patients indicates that CD38^+^ leukemic cells constitute a heterogeneous population including a small fraction of cells with an increased proliferative potential, ranging from 0.08 to 1.7% (143), suggesting that not all CD38^+^ cells are actively proliferating.

Pepper et al. also characterized the CD38^+^ fraction showing that CD38^+^ CLL cells overexpressed vascular endothelial growth factor and its expression associated with increased expression of the anti-apoptotic protein MCL-1 (160). Overall, these data describe an interesting subset of proliferative CLL cells with overexpression of different molecules that suggest microenvironment signaling activation but are not enough to specifically identify the proliferative subset in CLL.

In a similar research line, Palacios et al. described a different, clonally related CLL subset with ongoing CSR to IgG (IgM/IgG), mainly found in progressive and U-CLL patients (161). This subpopulation exhibited increased levels of Ki-67 and Survivin proteins and decreased levels of p27^−Kip1^, which underline a clear proliferative behavior for these cells (145). Other molecular markers associated with tumor proliferation and microenvironment activation such as high expression levels of mRNA *of c-myc, Bcl-2, CD49d, Ccl3*, and *Ccl4* were found in this subset. Interestingly, Palacios et al. also found that AID, a molecule responsible for SHM and CSR of Ig genes in B lymphocytes, is expressed mainly in this proliferative subpopulation of the CLL clone (161). Since AID is a mutagenic enzyme, its expression is strictly regulated in B cells (162) and in physiological conditions limited to the centrocyte/centroblast stages in germinal centers (163). The discovery that CLL cells with ongoing CSR expressed AID in the PB of progressive CLL and that percentages of these subset ranged between 0.1 and 5% leads researchers to speculate that these cells could also represent a proliferative compartment of the CLL clone (145).

It is difficult to determine the precise role of these highly proliferating, activated leukemic B cells expressing class switched Igs distinct from that of the parent clone. Since the presence of this subset is clearly associated to poor prognosis, it might play an adjuvant role in the maintenance of the CLL proliferative pool. However, given their increased proliferative potential, one would expect that they would eventually outnumber the IgM^+^ cells, and this is not the case. Thus, we assume that these cells undergo apoptosis once leaving the PCs. A recent study suggesting a link between AID expression and B-cell apoptosis in GC favors this view (164). In these conditions, the IgM^+^/IgG^+^ subset could reflect the existence of an active microenvironment leading to permanent stimulation of the IgM^+^ pool, which could turn on the CSR machinery maintaining this subset in the PB. Despite the fact that a clear association was demonstrated between the existence of this subset with an unmutated profile and poor clinical outcome, their roles in CLL evolution remain to be elucidated.

Subsequently, another proliferative subset that can also express AID was identified by Calissano et al. (165). They characterized the proliferative and resting compartments of CLL using differences in the densities of a surface membrane molecule upregulated after normal B-cell activation, CD5 (166), and another involved in maintaining B-cell retention in contact with stromal elements of solid lymphoid tissues, CXCR4 (165). Specifically, they postulated that high CD5 density would reflect cellular activation as in normal human B cells and low CXCR4 levels would identify cells that internalized the receptor because of an activation event and thereby moved out of a lymphoid tissue to the periphery (165).

This work proposed a life cycle for individual CLL cells representing a continuum between the CXCR4^dim^CD5^bright^, CXCR4^int^CD5^int^, and CXCR4^bright^CD5^dim^ fractions. At one extreme is the proliferative fraction, highly enriched in young, vital cells that recently left a solid lymphoid tissue where activation and proliferation occurred. At the other end is the resting compartment, containing older, less robust cells that may have been circulating in the periphery longer and are attempting through high CXCR4 levels to migrate into a solid tissue niche to avoid death (165).

A similar approach involving the marker CXCR4 was performed by Huemer et al., using another B cell activation marker CD86 to identify the proliferative fraction (167). CD86 is upregulated in B lymphocytes undergoing cell division in the light and dark zones of the germinal centers (168) and, in line with this idea, they found that expression of the proliferation associated antigen Ki67 was higher in CD86^high^CXCR4^low^ CLL cells than in CD86^low^ CLL cells (167). This proliferative fraction also expressed AID and overlapped with the CXCR4^dim^CD5^bright^ subset previously described (165).

There is clear evidence that a fraction of the CLL clone is generated each day. These results and those of Herishanu et al., describing that the LN constitute a privileged site of tumor proliferation (148), underlining the importance of the proliferating fractions inside the tumor clone of different CLL patients. Being aware that AID overexpression is a hallmark of these subsets and is associated with loss of target specificity resulting in mutations in non-Ig genes (*Bcl-6, Myc, Pax-5*, and *Rhoh*) (169), it is logical to assume that progressive disease could be related to clonal evolution of these proliferating cells. The constitutive expression of AID in the leukemic clone history could be a key event in disease progression (Figure 2).

## Activation-Induced Deaminase in CLL Cells and Its Effects on CLL-Cell Biology

### AID Molecular and Structural Considerations in CLL

There are no reports suggesting that the *aicda* gene is mutated in CLL, as is the case in Hyper-IgM Syndrome, type 2. However, the splice variants mentioned above that have been found in normal B lymphocytes have also been detected in CLL by our (124, 125) and other (25, 123, 170–172) laboratories. Furthermore, like normal human B lymphocytes, CLL B cells express only a single variant form of AID mRNA (25), although this might change with environmental input. Thus, it appeared that splice variants lacking the CSR domain were better able to carry out SHM than full-length protein. This was consistent with the finding that normal germinal center B lymphocytes express full-length AID mRNA, whereas the CSR-deficient, SHM-enhanced spliced variant was present at higher levels in CLL B cells. Interestingly, AID transcript levels have been associated with the occurrence of CLL founding events. In a cohort of 149 patients, higher levels of alternatively spliced transcripts of AID (AIDΔE4a, AIDΔE4, AIDivs3, and AIDΔE3E4) were associated with trisomy of chromosome 12. Functional analysis of AID splice variants revealed loss of their activity with respect to SHM, CSR, and induction of double-strand DNA breaks (25, 172).

Despite these findings, a recent study suggested that AID splice variant proteins are not functional (172), which might be expected since AID splice variant patterns are not different at the time of diagnosis, nor do they appear to have an impact on progression-free and overall survival (171). Favoring this hypothesis, the work of Rebhandl et al. proposed that despite the presence of alternatively spliced AID transcripts, only full-length AID was detected at the protein level (173). Interestingly, when analyzed under limiting dilution conditions, it became clear that AID was expressed in a very minor subpopulation of the CLL clone (125). As has been previously demonstrated, the expression of AID in CLL is restricted to the proliferative fractions, which can be visualized in the peripheral blood of the most progressive cases (88, 174).

### Molecular Actions and Targeting of AID in CLL

AID efficiently mutates the V and S regions of CLL cells. Since some CLL cells do not contain any or only a few mutations (U-CLL) (134, 135) but can express isotype-class switched Igs (175, 176), we correlated AID levels with V and S region mutations in CLL patients (124). This revealed that U-CLL cells express AID, and this expression associates with mutations in the S region. The cells of these patients also contained clonally related, isotype-switched transcripts (124), as previously reported (176–178).

Next, we focused on mutations occurring in the IgHV gene to determine those that could be attributed to the action of AID (158). Moreover, replacement (R) mutations segregated to complementarity-determining region 1 (CDR1) and CDR2 and silent mutations concentrated more in framework regions, FR (CDR R:S = 4.60 vs. FR R:S = 1.72). These results were consistent with the IgHV mutations resulting from an AID-mediated process and being selected for altered but structurally sound BCRs. Similar findings were reported by others (179), who also investigated expression of DNA polymerases zeta and eta that can be involved in repair of DNA altered by AID.

Because overlapping AID hotspots are critical sites for V region diversification (61) and key evolutionary components of human IgHV genes (62), Yuan et al. analyzed those IgHVs most used in CLL patients and in the clinically relevant U-CLL and M-CLL subgroups for such hotspots (180). This revealed a highly significant, but surprisingly inverse relationship between the number of WGCW hotspots in the germline IgHV and the observed mutation frequency in patients. This correlation was not observed in sequences from the B-cell repertoires of normal individuals and from those patients with autoimmune diseases. The relevance of these observations to the development of CLL and to patient outcome is not clear at this point.

### Relationship of AID Expression to CLL Cell Activation

There is consensus that AID activity is upregulated in activated B lymphocytes in G1 or the G1-S phase transition and is maximally expressed in the highly proliferative centroblasts in the germinal center. Thus, proliferating leukemic cells resemble this situation and upregulate or maintain AID expression (50). When analyzing PCs in lymph nodes for the presence of AID protein, we found that only Ki-67^+^ leukemic B cells contained the enzyme (88). Moreover, when examining CLL cells in the blood, we (124) and others (123, 170) found that AID is present in subsets of CLL clones, in particular those of the U-CLL type. However, expression was restricted to a minor subfraction of the clone, ~0.1% (125, 161).

Based on the latter observation and the requirement for cell cycle entry to upregulate AID, we tested if the AID^+^ fraction in CLL clones correlated with those few CLL cells in the blood that contain ^2^H-DNA after ingestion of ^2^H_2_O (165, 181). Cells containing ^2^H-DNA have recently completed the cell cycle and duplicated their DNA (182). In CLL, this fraction is small, ranging from 0.1 to ~4.0% of the CLL clone on a daily basis (156, 157, 165, 181). Indeed, we found that recently divided cells (“proliferative fraction”), identified by a CXCR4^Dim^CD5^Bright^ (165) or CLL cells undergoing CSR to IgG (IgM^+^/IgG^+^ phenotype) (161), were the only circulating leukemic cells that expressed AID (88, 145, 161). Cells that had divided earlier did not. Thus, those few cells in the blood of CLL patients that express AID are recent emigrants from tissue niches where cell division occurred and where AID was upregulated. Therefore, although circulating CLL B cells are unlike normal B lymphocytes from healthy people in that they express AID, this expression is a function of upregulation as a consequence of proliferation in tissue niches and not an aberrant function of a neoplastic cell (Figure 2).

Moreover, AID expression is dynamic, with its levels and the cells making it changing over time in individual patients (88, 125). This recognition led us to follow the clinical course of >100 patients over several years (88). The presence of circulating AID^+^ cells presaged significantly shorter time to first treatment and to decreased overall survival. This was the case, not only for U-CLL patients who had higher levels of AID, but also for the fraction of M-CLL patients with increased AID amounts. Moreover, U-CLL patients with high AID levels had a shorter overall survival than U-CLL patients with lower levels. Finally, AID levels correlated with cytogenetic abnormalities that associate with poor clinical outcome, and together, these foretold shorter time to treatment (88). The latter findings are reminiscent of other studies associating AID and AID^+^ cells with genomic abnormalities and clinical poor outcome (167, 170, 183–185).

#### Ibrutinib, Which Blocks CLL Cell Replication, Inhibits AID Expression

Ibrutinib has emerged as a potent treatment for CLL (186). Although the drug alone does not appear to be curative, it clearly delays disease progression and markedly improves patient quality of life (186). Because ibrutinib has been shown to rapidly inhibit CLL B cell proliferation *in vitro* (187, 188) and *in vivo* in mouse (188) and man (189), we tested if it would affect the size of the recently divided, proliferative fraction and thereby block AID expression (190).

In this way, we found that CLL proliferative fractions, defined as cells bearing a surface membrane phenotype of CXCR4^Dim^CD5^Bright^ (165) or IgM^+^IgG^+^ (161), were significantly decreased by ibrutinib. We recently demonstrated that ibrutinib downregulated AID in treated CLL patients and that, interestingly, this downregulation correlated with reduced AKT pathway and Janus Kinase 1 signaling (190). These findings also had important clinical implications since they showed that ibrutinib did not lead to increased levels of AID and thus would likely not result in genomic instability, as had been reported in a preclinical study (191).

Additionally, we studied the effects of ibrutinib on CLL cell growth directly in patients, using the ^2^H_2_O ingestion technique to label leukemic cells dividing *in vivo* (189). Treatment-naive patients with progressive disease, who were deemed to require treatment within 6 months, drank ^2^H_2_O before starting therapy, allowing the direct determination of birth rates of their leukemic clones and calculation of death rates based on blood counts.

Upon starting ibrutinib, birth rates decreased to negligible levels and death rates increased. This was direct documentation in patients that ibrutinib blocks CLL cell proliferation and that it promotes CLL cell death by inhibiting trafficking to tissue survival niches (189). Although AID levels were not measured in these samples, based on our data above and the knowledge that AID requires cell proliferation to be expressed, it is highly likely that AID's synthesis and its on- and off-target actions were aborted.

Collectively, therefore, these findings indicate several key features of AID expression in CLL. First, like normal B lymphocytes, AID is regulated by the stage of cell activation of the leukemic B cell, and those CLL cells in the blood expressing AID have recently divided and emigrated from lymphoid tissues, most likely lymph nodes based on the documented higher rate of cell division at that site (147). Second, the fraction of activated, AID^+^ cells is small and changes over time. Finally, the presence and levels of AID^+^ intraclonal members correlate directly with worse clinical course and survival.

Thus, the small AID^+^ fraction, which is contained almost exclusively in the recently divided proliferative fraction of a CLL clone and that just replicated its DNA, has the highest likelihood of having just developed a new genetic lesion that could lead to disease progression. Hence, this fraction is especially dangerous. This supposition is supported by our finding that the daily rate of CLL-cell division, which correlates with the extent of AID expression, correlates with poor prognostic markers (unmutated IgHV, levels of ZAP70 and CD38) (143, 192) and predicts time-to-first treatment (192). This helps explain the underpinnings that allow ibrutinib and other small molecules that inhibit the activation of CLL cells to extend and improve the quality of life of CLL patients (193). Another promising possibility to accomplish this is the use a bispecific antibody that in preclinical studies preferentially targets this small intraclonal fraction (194).

Other interesting drugs targeting PF cells expressing AID are the inhibitors of HSP90. Chaperon HSP90 regulates numerous signaling proteins and pathways helping the cancer survive environmental stresses (195). Results reported by Ortehwein et al. propose AID as a novel HSP90 client, and consequently, treatment with HSP90 inhibitors could inhibit AID nuclear import or induce AID degradation (196). Preliminary results in collaboration with Di-Noia lab allowed us to provide proof-of-concept that HSP90 inhibitors target human AID in primary CLL cells (197). Other studies have also proposed the use of HSP90 inhibitors as candidate drugs in CLL to achieve a multi-targeting effect by inhibition of AKT and different kinases signaling (198). Currently, clinical trials using Hsp90 inhibitors in CLL are under development. For example, SNX-5422 (a highly selective, small molecule inhibitor of the HSP90) in combination with Ibrutinib is being tested in a phase I clinical trial in CLL patients with residual disease (NCT02973399, by Esanex Inc.).

Thus, targeting the proliferative fraction appears to have therapeutic value in CLL, but more time is required to corroborate the success of this idea.

#### Microenvironmental Signals That Induce AID Expression in CLL B Cells *in vitro*

Like normal B lymphocytes, CLL cells upregulate AID in response to T-cell-dependent and T-cell-independent stimulation. Specifically, culturing CLL B cells with activated T lymphocytes (199); C3d-coated Ag, IL-4, and BAFF (200); CD40L + IL-4 (88, 124, 201); and TLR9 agonist + IL-15 (202) can lead to AID expression. Consistent with TLR signaling being relevant to this disease are gene expression analyses indicating that CLL cells in LNs express a profile consistent with TLR activation (203).

Specifically, we have provided T-cell-dependent stimulation in the form of intact activated T cells or T-cell-derived signals (CD40L + IL-4) to stimulate upregulation of AID and induce CSR (124) and SHM (88, 199) *in vitro*. In this way, we found that cultured CLL samples expressed AID mRNA and that production of AID protein varied considerably on a patient basis with the percentages of AID^+^ cells ranging from ~1 to ~80% (88). As expected, production of AID followed the extent of cell divisions undergone by a given sample. Furthermore, the cells in these cultures exhibited features consistent with the protein being biologically active, in that phospho-histone H2A.X (pH2A.X) that binds to dsDNA breaks, mRNA transcripts for IgG along with surface membrane IgG, and new DNA mutations in IgHV-D-J DNA sequences were found. Strikingly, there was no major difference between U-CLL and M-CLL cases for these findings. Finally, we investigated the association of AID expression with time to first treatment over an 8 year period. This revealed that patients with AID^+^ cells in the blood had significantly shorter time to treatment and overall survival (88).

Consistent with this, when primary CLL cells were transferred into lymphoid mice, a technique that leads to leukemic B-cell engraftment and growth (204), a relatively large fraction of transferred leukemic B cells synthesized AID and underwent CSR and SHM (205). Thus, the production of AID in response to T-cell co-stimulation can occur *in vivo* as well. Notably, the ability to express AID and undergo CSR and SHM *in vitro* and *in vivo* is as efficient for U-CLL clones as M-CLL clones (88, 205). This suggests strongly that the absence of IgHV-D-J mutations in U-CLL patients is not inherent but influenced by the microenvironment (205).

#### After Leukemic Transformation, AID Continues to Act on Ig Variable and Switch Regions in Individual Members of CLL Clones

Early studies analyzing molecular clones by Sanger sequencing suggested that CSR (201) and SHM (199, 206) occurred after leukemic transformation in individual, clonally related members of the leukemic CLL cell. This finding has been confirmed using next-generation, deep DNA sequencing (207).

Collectively, these data indicate that the AID mutational process continues after leukemic transformation within individual members of CLL clones, including those in which the clinically defined clone is classified as U-CLL. Thus, it is reasonable to expect that non-Ig genes could also be affected by this ongoing mutational process. The contributions of AID-mediated and non-AID-mediated mutations to genomic instability and disease progression are discussed in detail below.

## The CLL Genome

The CLL genome is characterized by the presence of structural alterations and a wide range of mutations that depicts a heterogeneous genomic landscape. IgHV mutation status and the presence of chromosome abnormalities are among the strongest predictors of clinical outcome (208). Approximately 80% of CLL patients carry at least one of four common chromosomal alterations, and the average CLL mutation rate ranges between 0.4 and 2.1 alterations per Megabase (48, 209). Overall, a typical CLL genome carries ~2,500 molecular lesions. M-CLL patients carry a higher mutational load (3,000 mutations/Mb) than U-CLL patients (2,000 mutations/Mb) (210).

### Structural Lesions

The most recurrent alterations in CLL are chromosome abnormalities, and the most frequent lesions are deletions of chromosome 13q (55% of cases), 17p (7%), 11q (6% to 18%), and trisomy 12 (12 to 16%) (211).

Structural rearrangements and SNV occur at similar frequencies when compared with other indolent B-cell lymphomas such as follicular lymphoma (212); however, their frequency is substantially lower than in most solid tumors. Initial studies of somatic copy number variations using karyotyping, fluorescence *in situ* hybridization, or single-nucleotide polymorphism arrays are extensively reviewed elsewhere (213).

In the clinical setting, these prevalent chromosomal aberrations are used in the widely accepted cytogenetic classification proposed by Dohner et al. (211). A hierarchical model based on five risk categories was established by correlating FISH lesions with clinical outcome. CLL cases with the 17p13 deletion (prevalence 7%) had the worst prognosis, independent of the presence of concomitant abnormalities, with a median survival of 32 months. Cases carrying the 11q22-q23 deletion (prevalence 18%) had a median survival of 79 months. Longer survival rates were associated with trisomy 12 (prevalence 16%, median survival 114 months), normal karyotype (prevalence 18%, median survival 111 months), and 13q14 monoallelic deletion (prevalence 55%, median survival 133 months) (211).

Cytogenetic lesions, however, do not entirely explain the genetic basis of the heterogeneous clinical outcome of CLL. The wide availability of next-generation genome sequencing has enabled the identification of new recurrent structural and single-nucleotide lesions. New recurrent genomic aberrations include trisomy 19, amplifications at 2p and 8q, and deletions at 8p, 6q21, 18p, and 20p (209, 214). A recent study supports the existence of multiple recurrent focal copy number alterations and of copy number neutral losses of heterozygosity affecting key oncogenic pathways, associated with higher proliferative capacity, shorter survival, and altered gene expression. Therefore, focal structural changes may also play a relevant role in CLL pathogenesis (215). Genome sequencing has also enabled the definition of the molecular correlates of CLL chromosomal aberrations (216, 217), in particular *Tp53*, the tumor suppressor gene affected by 17p13 deletion, and *Atm*, the gene targeted by 11q22-q23 deletion (218).

### Recurrent Oncogenic Mutations

Although recurrent chromosomal abnormalities can be found in most CLL patients, only very few single-nucleotide variants show a recurrence higher than 5% across patients. Additionally, a large number of biologically and clinically uncharacterized genes are mutated at a lower frequency.

Similar to the inherent clinical heterogeneity, the genetic landscape of CLL is markedly complex with a rapidly growing list of genes mutated at a low frequency. Most gene mutations still require rigorous validation in large, prospective patient studies, and only a few genes have been implied to have diagnostic and prognostic impact.

Within this heterogeneous landscape, there is a set of shared genetic lesions among B-cell malignancies, affecting similar mechanisms and processes such as DNA repair and antigen receptor signaling indicating certain degrees of shared pathways involved in lymphomagenesis (219, 220).

The most frequent recurrent mutations in CLL affect *Notch1, Sf3b1*, and *Birc3* and have been reported to occur in approximately 2–10% of patients within a general practice setting (221). The frequency of mutations of candidate driver genes, with the exception of *Myd88* and *Igll5*, has been consistently associated with progressive, high-risk disease or U-CLL (Table 1) (225, 226).

**Table 1:** Recurrently Mutated Genes in Different Clinical Settings.

	**Early (Binet A) (222) (%)**	**Advanced (Binet B/C) (222) (%)**	**Mutated (223) (%)**	**Unmutated (223) (%)**	**Refractory Relapsed (224) (%)**
NOTCH1	6	13	7.0	20.4	14.9
SF3B1	6	18			28.1
TP53	8	17			22.8
BIRC3	1.9	4.5			
MYD88	2	2.5	5.6	0.8	2.6
XPO1			0	4.6	14.9
KLHL6			4.5	0	
ATM					26.3

### The Role of AID in CLL Mutagenesis

SHM represents an endogenous mutator mechanism in B lymphocytes initiated by AID, and its mutagenesis has been associated with lymphomagenesis in B-cell neoplasms (195, 220). The availability of next-generation sequencing and the development of modern machine learning algorithms to deconvolute underlying mutagenic processes has enabled identification of putative mechanisms driving genetic lesions in cancer cells (227). In CLL, the main mechanisms consistently identified in patients are aging, enzymatic deamination, and defects in DNA repair (48, 227–229). Whereas, aging-induced deamination may account for up to three quarters of the single-variant substitutions, the remaining lesions can be linked to endogenous deamination and defects in DNA repair (48).

One intriguing issue regarding AID activity in lymphomagenesis is the apparent decoupling between AID expression and SHM, not only in CLL (124) but also in other B-cell malignancies such as follicular lymphoma (230, 231). In line with these results, the ongoing AID activity is enriched in higher risk U-CLL cases (228), and the contribution of subclonal aberrations to CLL pathogenesis is being increasingly recognized (232). The analysis of clonal and subclonal mutations has allowed the reconstruction of tumor phylogeny (233). Clonal lesions, which encompass mostly structural changes, generally correspond to earlier driving events, while subclonal lesions in driver genes (e.g., *Notch1* and *Sf3b1*) are acquired later over the course of the disease (222, 234, 235) (Table 1).

Considering all this evidence, it can be hypothesized that AID first plays a broader role at early stages of leukemogenesis contributing to the induction of founding events and later preferentially acts in proliferating fractions contributing to mutagenesis, facilitating the emergence of new clones involved in tumor progression.

An interesting study by Kasar et al. showed that in a cohort of 30 indolent CLL cases, c-AID and nc-AID signatures accounted for 25% (5 and 20% respectively) of somatic mutations (210, 236). Our preliminary data analyzing an unbiased CLL cohort (237) also shows a proportion of c-AID signature distribution similar to those described by Kasar and Brown (236). However, when follicular lymphoma (FL) cells (a germinal center B-cell malignancy with constitutive AID expression) are analyzed, our results revealed a c-AID contribution up to 9% (237).

Given that from a biological point of view CLL can be separated into two broad subsets according to the IgHV mutation status (208), we speculate that c-AIDl and nc-AID signature contribution could be different between both entities. U-CLL and progressive patients might have a more important ongoing c-AID signature similar to those presented in FL cells, whereas indolent and mutated CLL cases might exhibit a lower ongoing c-AID signature. At present, the real rate of the c-AID contribution in those progressive CLL patients in which a functional AID was established (25, 124) or in the CLL proliferative fractions that express AID in these patients (88, 161) remains unknown. Indeed, how this ongoing c-AID activity influences the mutational status of the CLL genome and, in consequence, in the long term, the disease outcome remains an important and unanswered question.

To move deep into this hypothesis, we recently developed an *in vivo* model by inducing constitutive AID expression in Eμ-TCL1 mice (named DT-AID). In this CLL-like model, we observed that DT-AID mice showed altered disease kinetics and higher percentages of CLL-cell proliferation, and these resulted in a more rapid progression of the disease compared with their TCL1 counterparts. Interestingly, a comparison of c-AID and nc-AID contributions between DT-AID and TCL1 revealed an increased ongoing c-AID signature in many non-Ig, cancer-related genes also described in human neoplasms (238).

In summary, cumulative evidence demonstrates that AID is involved in leukemogenesis and tumor progression. We speculate that AID is involved in mutagenesis of CLL clones at very early stages, thereby participating in founding events leaving a genomic footprint readily sizable by somatic signature analysis. At later stages or after cytotoxic treatment, AID could further act at the subclonal level, facilitating the emergence of additional, mutated malignant cells involved in progression.

## Author Contributions

PO and NC: conception, design, and writing the manuscript. PO, MN, and NC: writing and/or revision of the manuscript. All authors contributed to the article and approved the submitted version.

## Conflict of Interest

The authors declare that the research was conducted in the absence of any commercial or financial relationships that could be construed as a potential conflict of interest.
